# Surface Density of the Spongy and Palisade Parenchyma Layers of Leaves Extracted From Wideband Ultrasonic Resonance Spectra

**DOI:** 10.3389/fpls.2020.00695

**Published:** 2020-05-29

**Authors:** T. E. G. Alvarez-Arenas, D. Sancho-Knapik, J. J. Peguero-Pina, Eustaquio Gil-Pelegrín

**Affiliations:** ^1^Instituto de Tecnologías Físicas y de la Información (ITEFI), Spanish National Research Council (CSIC), Madrid, Spain; ^2^Unidad de Recursos Forestales, Centro de Investigación y Tecnología Agroalimentaria de Aragón (CITA), Zaragoza, Spain; ^3^Instituto Agroalimentario de Aragón – IA2 (CITA-Universidad de Zaragoza), Zaragoza, Spain

**Keywords:** ultrasound, air-coupled ultrasound, resonant spectroscopy, plant leaves, layered plant tissue, leaf’s parenchyma layers

## Abstract

The wide band and air-coupled ultrasonic resonant spectroscopy together with a modified Simulated Annealing metaheuristic algorithm and a 1D layered acoustic-model are used to resolve the structure of plant leaves. In particular, this paper focuses on the extraction of the surface density of the different layers of tissue in leaves having a relatively simple structure. There are three main reasons to select the surface density as the focus of this study: (i) it is a parameter directly extracted by the proposed technique and it requires no further processing, (ii) it is relevant in order to study the dynamic of the water within the different tissues of the leaves and also to study the differential development of the different tissues, and (iii) unlike other parameters provided by this technique (like resonant frequency, impedance, ultrasonic elastic modulus, or ultrasonic damping), this parameter can be easier to understand as it is a direct measure of mass per unit surface. The selection of leaves with a simple structure is justified by the convenience of avoiding an unnecessary complication of the data extraction step. In this work, the technique was applied to determine the surface density of the palisade and spongy parenchyma layers of tissue of *Ligustrum lucidum*, *Vitis vinifera*, and *Viburnum tinus* leaves. The first species was used to study the variation of the surface density at full turgor with the thickness of the leaf, while the two other species were used to study the variation of the surface densities with the variation in the leaf relative water content. Consistency of the results with other conventional measurements (like overall surface density, and cross-section optical and cryo-SEM images) is discussed. The results obtained reveal the potential of this technique; moreover, the technique presents the additional advantage that can be applied *in-vivo* as it is completely non-invasive, non-destructive, fast, and equipment required is portable.

## Introduction

The wide-band, air-coupled ultrasonic resonant spectroscopy method has been presented by Álvarez-Arenas et al. (2018). This method is an extension of the air-coupled ultrasonic resonant spectroscopy method presented in Álvarez-Arenas et al. (2009); Sancho-Knapik et al. (2010, 2012, 2013a) that has been widely used for different applications (Farinas and Alvarez-Arenas, 2014; Farinas et al., 2014) where the determination of leaves water content attracted most of the interest: (Álvarez-Arenas et al., 2016; Sancho-Knapik et al., 2016; Farinas et al., 2019).

This ultrasonic technique is one of the methods available to measure physical properties of plant leaves. Other techniques are based on the measurement of dielectrical properties (Repo, 1988; Zhang and Willison, 1993; Chuah et al., 1995; Mizukami et al., 2006), the leaf response to pressure (Zimmermann et al., 2008; Rueger et al., 2011), and the leaf response to microwaves (Martínez et al., 1995; Menzel et al., 2009; Sancho-Knapik et al., 2013b). The main features of the ultrasonic method is that it is completely contactless, non-invasive, and non-destructive and that it can be applied *in-vivo*. In addition, the novel wide band ultrasonic technique present the unique feature that it can obtain information about the different layers in the leaves, also in a completely non-invasive and non-destructive way, which allows us to study not only the overall leaf properties and their variations, but also the differences between the main layers of tissue in the leaves and the differences in the variation of their properties (for example with the leaf development or with the modification of the leaf water content).

In a few words, the ultrasonic method consist on using a pair of air-coupled ultrasonic transducers (transmitter and receiver) whose frequency band is tuned to include the fundamental resonant frequency of the leaf thickness mode. The transmission coefficient spectra of the leaf around this fundamental resonant frequency is measured and leaf properties like thickness, density, impedance, elastic modulus, and ultrasonic damping are extracted from the solution of the inverse problem using a 1 dimensional model and assuming an effective medium approach to acoustically model the leaf. The use of this extremely simple model is justified by the following facts: (i) at the first thickness resonance the wavelength equals two times the thickness of the leaf, so it is much larger than the inner details of the leaf structure, (ii) the calculated spectra reproduce quite well the measured behavior, and (iii) the physical and physiological meaningfulness of the extracted parameters were confirmed by comparison of the extracted parameters with leaf properties obtained by conventional means (like total thickness, overall density, water potential, or turgor loss point).

By increasing the frequency it is possible to observe several orders of the leaf thickness resonances. However, those leaf spectra showing two or more resonances revealed a clear harmonic distortion. The effective medium approach used before, where the leaf is modeled as a homogeneous layer, is unable to provide a good fit into the measured spectra in these cases. This was not a fully unexpected result as the increase of the frequency implies a reduction of the wavelength, so the ultrasonic wave becomes more sensitive to the details of the leaf inner structure, especially to the layered structure. Álvarez-Arenas et al. (2018) shows that a simple layered model, where an effective acoustic model of the leaf is built using two layers (that mainly correspond to the spongy parenchyma -SP- and the palisade parenchyma -PP-), is able to explain or reproduce the measured spectra and that the extracted data (like impedance, resonant frequency, and elastic modulus) are consistent with direct measurements and with cross-section SEM observations of the leaf structure.

The two main challenges of the use of the wide-band technique are: (i) the solution of the inverse problem with two layers becomes intractable by conventional means and (ii) the difficulty of having efficient air-coupled ultrasonic transducers able to cover the required frequency range. For the former, a metaheuristic approach that consists on a modification of the Simulated Annealing algorithm was proposed for this application and successfully used in Álvarez-Arenas et al. (2018), for the later, the technology developed by CSIC to produce efficient air-coupled ultrasonic transducers with wide band response (Álvarez-Arenas et al., 2018) have been employed.

The first case of study shown in this work is the extraction of the surface densities of the PP and SP layers of tissue of *Ligustrum lucidum* leaves. For this case, leaves of different thickness (300–800 micron) have been used. Álvarez-Arenas et al. (2016, 2018) have shown that these leaves can be well measured with this technique and that the increase of the leaf thickness is mainly produced by an increase of the thickness of the PP layer. Therefore, this species provides a convenient case of study.

The second application of this technique presented here corresponds to the extraction of the surface densities of PP and SP layers in *Viburnum tinus* and *Vitis vinifera* leaves with different levels of water content, from full turgor down to the turgor loss point and beyond. As the loss of water will produce a decrease of the surface density, it is interesting to use this parameter to evaluate the water dynamic and use it as a proxy of relative water content (RWC). Moreover, the possibility to have measurements of different tissues of the leaf is extremely attractive as this information can be used to understand the different ways these tissues might cope with water deficit and how the whole leaf could manage these differences.

## Materials and Methods

### Plant Material

Three plant species were used in this study: *Ligustrum lucidum* W.T. Aiton, *Viburnum tinus* L., and *V. vinifera* L. Plant material of these species was collected from the Real Jardin Botánico of Madrid (CSIC) following the next procedure. South exposure shoots from well-watered specimens were harvested early in the morning, introduced in sealed plastic bags together with wet filter paper to preserved leaf full turgor, and carried immediately to the laboratory in a portable cooler. Once in the lab, full-developed mature leaves without defects were selected and immediately used for the measurements. This procedure was repeated every single day when measurements were performed.

Concerning *L. lucidum*, 70 leaves with different thickness (from 250 to 800 microns) were measured throughout 5 days to study the influence of the thickness on the surface density of the different tissue layers. Each day, we selected 10 leaves for ultrasonic measurements and other four leaves to obtain the overall leaf surface and volumetric density and the images of the leaf cross sections. For *V. tinus*, a total of 56 leaves were measured throughout 8 days, selected in groups of 7 leaves per day (5 of them for ultrasonic and 2 for the other measurements). Finally, for *V. vinifera*, we have measured 120 leaves throughout 20 days, selected in groups of six leaves per day (4 for ultrasonic and 2 for the other measurements).

### Ultrasonic Methods

Two pairs of custom air-coupled transducers developed at CSIC (Alvarez-Arenas, 2004, 2017) were used to measure the transmission coefficient over a frequency band wide enough to observe, at least, two orders of the leaves thickness resonances. One pair with center frequency at 0.3 MHz (usable frequency range 0.15–0.5) and the other at 1.00 MHz (usable frequency range 0.5–1.6 MHz). Transducers (transmitter and receiver) were mounted on an *U*-shaped holder, that kept them (transmitter and receiver) in opposition and at a distance of 60 mm and 40 mm for the 0.3 MHz and the 1.0 MHz transducers, respectively. Aperture of the transducers is circular with diameter of 20 and 15 mm for the 0.3 and the 1.0 MHz, respectively. Area of the leaf were the ultrasonic measurements are performed is similar to transducer aperture. In addition, a polycarbonate cover wrapped around the *U*-shaped holder protects measurement area and provides a groove to introduce the leaves. This groove permits to properly place the leaf in between the transducers: at normal incidence and at the middle point. Images of this device can be seen in Álvarez-Arenas et al. (2016, 2018). Transmitter transducer is driven by a negative semicycle square wave, amplitude 400 V provided by an Olympus pulser/receiver (PR5077). The received signal in the receiver transducer is connected to the receiver stage of the PR5077, where it is amplified (0 dB for the reference signal: no leaf between transducers, and 40 dB for the measured signal through the leaves), and then sent to a digital oscilloscope Tektronix 5054. The oscilloscope is triggered by the synchronism signal provided by the PR5077. The oscilloscope digitizes the signal, averages it 100 times and sends it to a PC to extract fast Fourier transform (FFT).

First, the signal at R× without any leaf between the transducers and at 0 dB gain is acquired and FFT extracted. This is used as calibration or reference value. Then the leaf is placed in between the transducers, gain set to 40 dB and the measurement repeated.

*Viburnum tinus* leaves and *L. lucidum* leaves were measured at two points in the wider part of the leaf, at both sides of the midrib and trying to avoid it. *V. vinifera* leaves were measured at three different locations matching main interveinal areas.

### Leaf Dehydratation Measurements

*Viburnum tinus* and *V. vinifera* leaves were left to dry at room conditions. Ultrasonic measurements were repeated every 5 min during 4 h. At each step, leaves were weighed (*M*). The first measurement corresponds to full turgor condition (*M*^*FT*^). After ultrasonic measurements were finished, leaves were placed in an oven at 80°C for 48 h. After this, leaves were weighed again to obtain the leaf dry mass (*M*^*dry*^).

**FIGURE 1:**
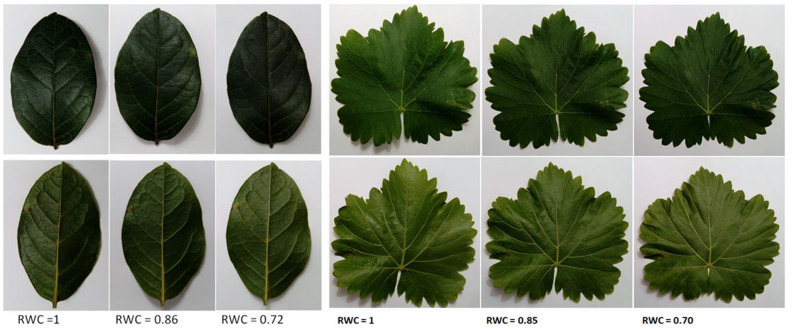
Pictures of leaves of *Viburnum tinus* (left) and *Vitis vinifera* (right) at different values of RWC. The adaxial part of the leaf is showed up, while the abaxial is showed down.

Figure 1 shows some pictures of one leaf of *V. tinus* and *V. vinifera* at different values of RWC. This figure shows that the deformation of the leaves do not compromise the integrity of the ultrasonic measurement as the flatness of the measured areas remains rather unchanged.

Relative water content at state “*i*” was obtained from:

(1) $$R\,W\,C^{i}=\frac{M^{i}-M^{d\,r\,y}}{M^{F\,T}-M^{d\,r\,y}}$$

It is of interest to show the close relationship between RWC and the surface density measurements. It can be easily derived:

(2) $$R\,W\,C^{i}=\frac{\rho_{S}^{i}\,\varepsilon^{i}-\rho_{S}^{d\,r\,y}\,\varepsilon^{d\,r\,y}}{\rho_{S}^{F\,T}-\rho_{S}^{d\,r\,y}\,\varepsilon^{d\,r\,y}}$$

where $\rho_{S}^{i}$ is the leaf surface density at state “*i*” and ε^*i*^ = *S^i^*/*S^FT^*, where *S* is the leaf surface, so ε^*i*^ is the leaf lateral shrinkage, that is ε ≤ 1. In the case that leaf lateral shrinkage can be considered negligible (ε = 1), then Eq. 2 reduces to:

(3) $$R\,W\,C=\frac{\rho_{S}-\rho_{S}^{d\,r\,y}}{\rho_{S}^{F\,T}-\rho_{S}^{d\,r\,y}}$$

which is completely analogous to Eq. 1.

### Leaf Thickness, Leaf Density, and Anatomical Images

Before each of the ultrasonic measurements, leaf thickness was measured using a micrometer. To obtain overall leaf surface density and volumetric density (ρ_*S*_ and *ρ*, respectively) two circles of 15 mm diameter were cut from each leaf by using a punch holder. Excised disks were weighed and thickness measured using a micrometer at 5 different locations to get an average value.

Both optical and cryo-SEM images of the leaves cross section were obtained for the three species at full turgor. In addition, cryo-SEM images of *V. tinus* leaves at different levels of water potential, measured by using a pressure chamber, were also obtained. Optical images of the leaf cross section were obtained by cutting the leaves into two cross-sections (0.2–0.3 mm apart) with a scalpel and the aid of a dissecting microscope. Then, a camera coupled to a light microscope was used to capture the leaf cross-sections which were later analyzed using ImageJ to obtain the total leaf thickness and the thicknesses of both palisade and spongy parenchyma. The purpose of obtaining cross-section images of the studied leaves was two-fold:

1. To show that the acoustic two-layers model for the leaves is a sensible one and to identify what is the relationship between the layers in the acoustic model and the actual layers of tissue in the leaves.
2. To get some insight into the modifications of these layers with leaf thickness (*L. lucidum*) and with water content (*V. tinus*), so that it is possible to infer some information about what modification can be expected in the surface density of the different layers.

### Leaf Parameters Extraction From the Measured Spectra: Solution of the Inverse Problem

All measured ultrasonic transmission coefficients of the studied leaves contain, at least, two orders of the leaf thickness resonances. This response can be theoretically calculated using a layered model for the leaf. The required parameters to compute the leaf transmission coefficients are the impedance of the medium where measurements are preformed (air in this case) and:

(4) $$f_{r\,e\,s}^{i},Z^{i},{\left(\alpha_{0}\,t\right)}^{i},n^{i};i=1,2,\ldots \,N$$

where:

*N* is the total number of layers in the acoustic model of the leaf, the superscript *i* denotes the number of layer, *$f_{r\,e\,s}^{i}$* is the resonant frequency of layer *i*, defined as *v*/*t* (*v* = ultrasound velocity and *t* = layer thickness), *Z*^*i*^ the complex acoustic impedance (*Z*_*R*_ + *jZ*_*i*_), $\alpha_{0}^{i}$ the ultrasonic attenuation coefficient and the parameter *n*^*i*^ describes the variation of the attenuation coefficient of the *i*th layer with the frequency as a power law:

(5) $$\alpha =\alpha_{0}\,{\left(\frac{f}{f_{0}}\right)}^{n}$$

Actually, *Z*_*R*_ and *Z*_*i*_ are not independent parameters, as it can be shown that:

(6) $$Z_{i}=\frac{Z_{R}}{f_{r\,e\,s}\,\alpha \,t}$$

The estimation of the deviation of the calculated transmission coefficient: *TC*[$f_{r\,e\,s}^{i},Z^{i},{\left(\alpha \,t\right)}^{i},n^{i}$] compared to the measured one: *TC*^*exp*^ is obtained using the L2 norm. The search for the set of parameters in Eq. 4 that minimize this deviation is performed using a modified Simulated Annealing algorithm and the procedure explained in Álvarez-Arenas et al. (2018).

Therefore, there are a number of parameters to be determined (dimension of the space of search) equal to 4 × N. The solution of this problem for one layer can be found with conventional techniques (refs), but for two layers, the number of parameters is eight and it becomes necessary to use a metaheuristic approach.

The selection of the number of layers to be used in the acoustic model for the leaf is selected, as explained in Álvarez-Arenas et al. (2018) on the basis of the following features:

1. Information in the measured spectra. For example, the number of resonances observed in the spectra. If there is only one (the first one) the measurement does not contain enough information to obtain more than four parameters. Moreover, a one-layer model has always been able to provide a good fitting.
2. Capability of the model to reproduce the measured response and the Occam’s razor principle. That is, if a one-layer leaf model is able to reproduce the observed behavior, this model is then preferred over the two-layers model and so on.
3. Compatibility between the acoustic model of the leaf and the actual leaf structure.

The algorithm is run five times for each case in order to have an estimation of the accurateness of the solution, to check for the existence of multiple solutions and to detect the presence of local minima.

The real part of the acoustic impedance (Z_*R*_) is given by: Z_*R*_ = ρ.*v* where ρ is the density of the medium and *v* the ultrasound velocity. Hence, it is clear that the surface density of the *i*-layer is then given by:

(7) $$\rho_{S}^{i}=\frac{Z_{R}^{i}}{f_{r\,e\,s}^{i}}=\rho^{i}\,t^{i}$$

Another interesting feature of $\rho_{S}^{i}$ is that:

(8) $$\sum_{i=1}^{N}\rho_{S}^{i}=\rho_{S}$$

where ρ_*S*_ is the leaf overall surface density that can be easily obtained by conventional means, so Eq. 8 can be used to validate the values of $\rho_{S}^{i}$ obtained with the ultrasonic technique.

## Results

### Cross-Section Images

**FIGURE 2:**
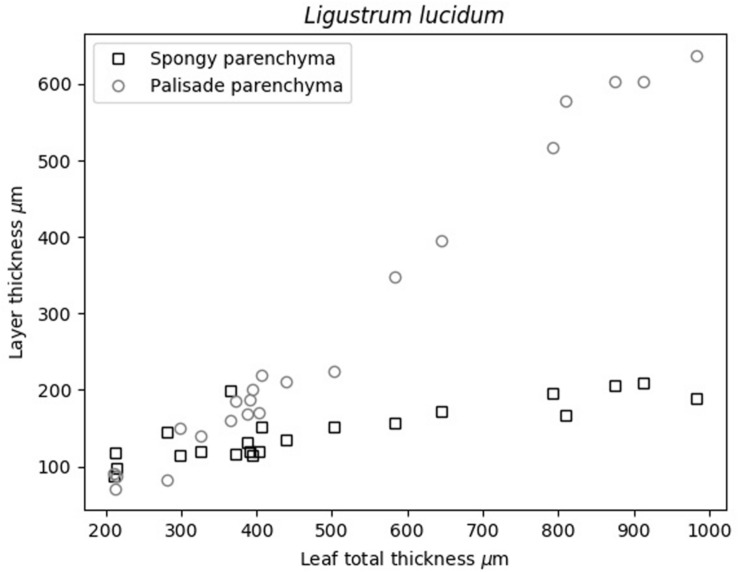
Variation in the thickness of the PP and SP layers with the total leaf thickness.

Previous analysis of cryo-SEM images of the cross-section of *L. lucidum* leaves were shown in Álvarez-Arenas et al. (2018). These images support the proposed acoustic layered model of these leaves, the use of two layers in the model, and the fact that leaf thickness increase is achieved by the thickening of the palisade parenchyma layer. Obtained thickness of the PP and SP layers in *L. lucidum* leaves are shown in Figure 2.

**FIGURE 3:**
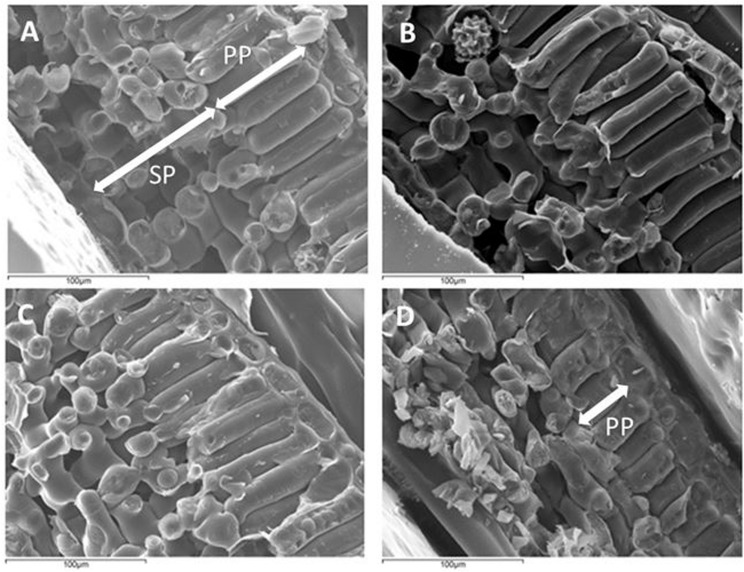
Cryo-SEM images of *Viburnum tinus* leaves cross section at full turgor **(A)** and at water potential of: –2.0 **(B)**, -2.6 **(C)** and -3.5 **(D)** MPa. SP: spongy parenchyma. PP: palisade parenchyma. Notice the reduction in PP thickness between **A** and **D**.

This layered structure of the leaves is also present in *V. tinus* leaves as it can be clearly seen in the cryo-SEM images (Figure 3A). For *V. vinifera* there are numerous evidences in the literature (see, for example, Alonso et al., 2011; Monteiro et al., 2013; Navarro et al., 2019). The two layer approach for the layer acoustic model is fully supported by the features observed in these images. This is because ultrasonic waves are only sensitive to variations of the acoustic impedance in the leaf. As the acoustic impedance is the result of the multiplication of the volumetric density and the ultrasound velocity, it is clear that the major acoustic differentiation within the leaf comes from the fact that the porosity in the SP layer is much larger and cell shape more rounded, hence, both density and ultrasound velocity in this layer must be much smaller. Additionally, cryo-SEM images also show a reduction in the PP thickness of *V. tinus* leaves toward lower values of water potential (Figure 3).

### Measured Resonance Spectra and Calculated Ones Assuming a Two-Layers Model and the Leaf Parameters Obtained From the Solution of the Inverse Problem

**FIGURE 4:**
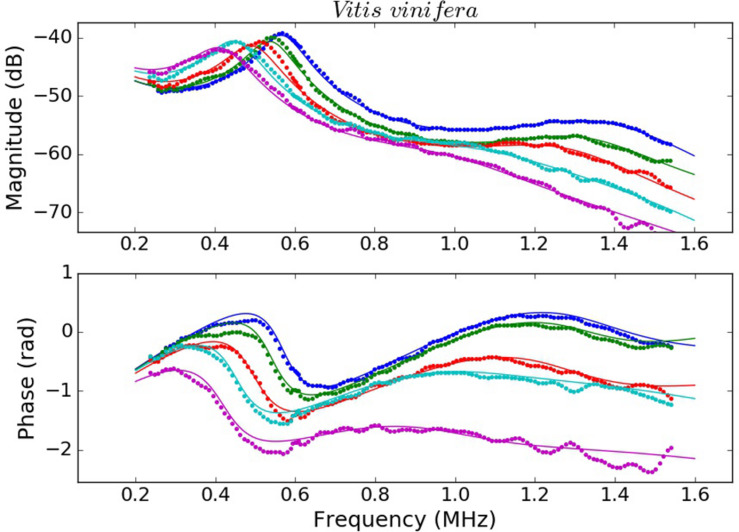
Measured (dots) and calculated (solid line) magnitude (top) and phase spectra (bottom) of *V. vinifera* leaves at different values of RWC 1 (blue), 0.96 (green), 0.93 (red), 0.87 (cyan), and 0.82 (magenta).

**FIGURE 5:**
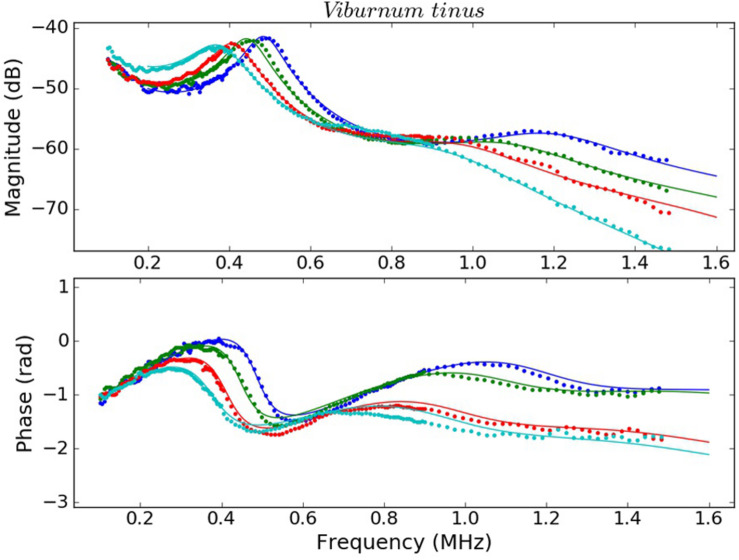
Measured (dots) and calculated (solid line) magnitude (top) and phase spectra (bottom) of *V. tinus* leaves at different values of RWC: 1.00 (blue), 0.96 (green), 0.91 (red), and 0.85 (cyan).

Examples of the measured spectra of *V. tinus* and *V. vinifera* leaves at different conditions (water content) and the calculated ones using the values extracted from the solution of the inverse problem using the Simulated Annealing algorithm are shown in Figures 4, 5. For *L. lucidum* leaves, measured and calculated spectra for different leaf thicknesses are shown in Álvarez-Arenas et al. (2018), and are, therefore, not repeated here. These results illustrate the main features of the measured spectra, their variation when leaves are modified and the goodness of the fittings. This later feature also reveals the capability of the proposed model to reproduce the actual leaf ultrasonic response.

### Variation of the Mass Per Unit Area in the PP and SM Layers of the Leaf With the Total Leaf Thickness: *L. lucidum*

**FIGURE 6:**
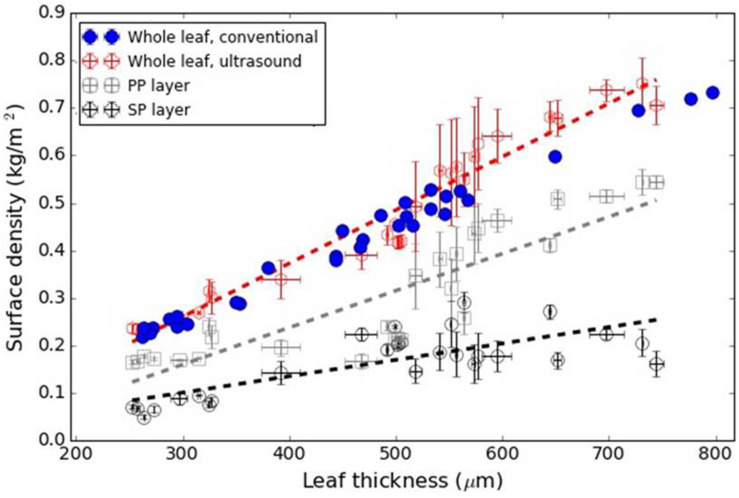
*L. lucidum*: Surface density of the PP and SP layers (from the ultrasonic technique) and overall surface density from Eq. 8 and direct measurements. Dashed lines represent a trend line just for visualization purposes.

Figure 6 shows the variation in the surface density of the PP and SP layers versus leaf thickness extracted from the ultrasonic measurements, the calculated overall surface density (from Eq. 8) and the value of the overall surface density obtained from the excised leaf circles using the circle surface and mass.

### Variation of the Mass Per Unit Area in the PP and SM Layers of the Leaf With the Leaf RWC: *V. tinus* and *V. vinifera*

**FIGURE 7:**
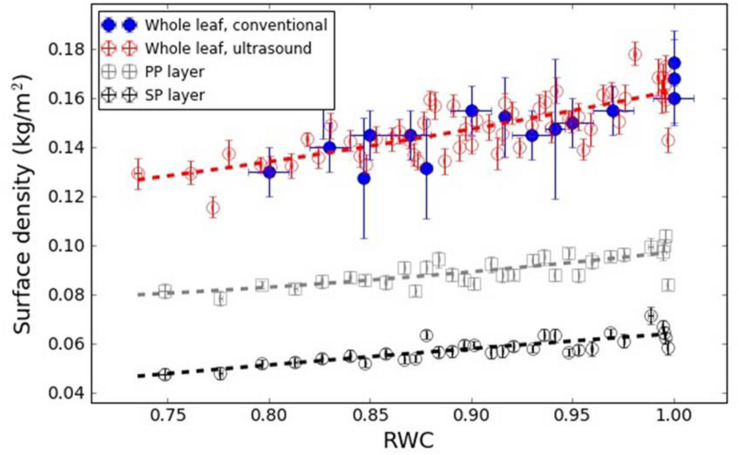
Surface density of the PP and SP layers and of the whole leaf versus RWC for the *V. vinifera* leaves. Dashed lines represent a trend line just for visualization purposes.

**FIGURE 8:**
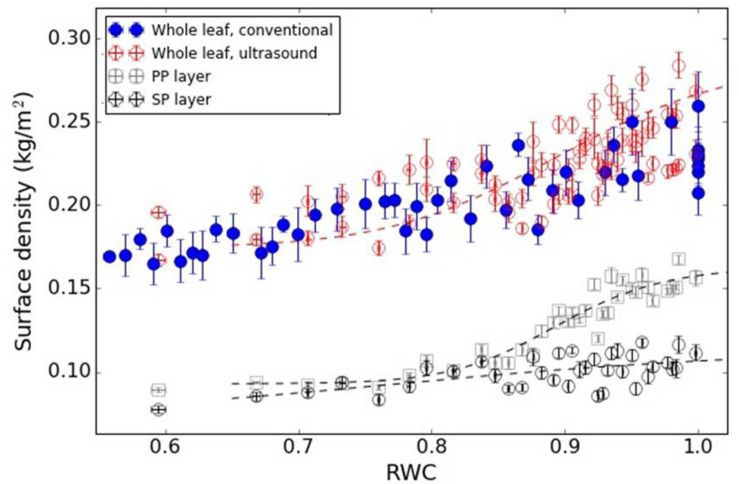
Surface density of the PP and SP layers and of the whole leaf versus RWC for the *V. tinus* leaves. Dashed lines represent a trend line just for visualization purposes (using a logistic function).

Figures 7, 8 show the variation in the surface density of the PP and SP layers and of the whole leaf with RWC for the *V. vinifera* and the *V. tinus* leaves.

## Discussion

This work shows that resonant ultrasonic transmission coefficient in the frequency range 0.15–1.6 MHz measured using air-coupled ultrasound at normal incidence in *V. vinifera* and *V. tinus* leaves present, at least, two orders of resonances and that the measured magnitude and phase spectra can be well reproduced theoretically using a 1D two-layered model. Moreover, a modified simulated annealing algorithm (Álvarez-Arenas et al., 2018) has proven to be efficient to solve the inverse problem and to extract the parameters of the layers by achieving and excellent fitting of the calculated spectra into the measured ones (Figures 4, 5, see Álvarez-Arenas et al., 2018 for *L. lucidum* results).

Cross-section images of the leaves suggest that the two layers in the acoustic leaf model correspond to the Palisade parenchyma + adaxial epidermis and the Spongy parenchyma + abaxial epidermis, as the major acoustic differentiation within the leaves is produced by both the larger porosity and the more rounded cell shape in the SP layer and the fact that there is a clear boundary between PP and SP layers. It can be worthwhile to remind that the acoustic differentiation between two tissues (two materials in general) is produced by the difference in the acoustic impedances. As the acoustic impedance is the product of density and ultrasound velocity, and ultrasound velocity depends on elastic modulus and density, then any difference in elastic modulus or density will contribute to variations in the acoustic impedance.

Focus of the paper is set on the surface density of these two layers extracted from the measured resonant spectra by the proposed modified Simulated Annealing algorithm and the 1D layered model for the leaves. As surface density is a measure of mass per area, this parameter can provide direct information about the variation of the water content or the biomass in the different tissues in the leaf. In addition, from the extracted surface density of the two layers in the leaf it is possible to obtain the total leaf surface density (Eq. 8), and this result can be compared with direct measurements obtained from excised circles from the leaves, and the measurement of area and mass. This can be used as an independent verification the obtained parameters.

Therefore, in all cases a direct comparison between total leaf surface density obtained by a conventional mean and extracted from the resonance spectra have been shown. Results appear in Figures 6–8 for *L. lucidum*, *V. vinifera* and *V. tinus*, respectively. In all cases, the achieved agreement between both (overall) surface densities estimations is within the experimental error. This is a first verification of the correctness of the leaf parameters obtained by the proposed approach.

Álvarez-Arenas et al. (2018) showed that *L. lucidum* leaves present the interesting feature that the leaf thickness increase is, mainly, produced by the increase of the thickness of the PP layer. Measurements in this work (Figure 2) verify and quantify this feature. This is of interest as this permit us to anticipate that variation in surface density of PP and SP layers with total leaf thickness, must behave in a similar way compared with the variation in the thicknesses of each layer.

Figure 6 shows the surface density of the PP and the SP layers of *L. lucidum* leaves extracted from the resonance spectra. In this case, leaves of different thickness, all of them at full turgor, have been measured. In all cases, the surface density is larger for the PP layer than for the SM layer, which is an expected result due to the fact that the thickness of the PP layer is always larger, or at least equal, and the porosity of the SM layer is higher. As expected from data in Figure 1 and cryoSEM images shown in Álvarez-Arenas et al. (2018) the increase of the surface density of the PP layer is more pronounced than the SM layer when the leaf thickness increases. This is a second verification of the correctness of the leaf parameters obtained by the proposed approach.

Figure 7 shows the surface density of the PP and the SP layers of *V. vinifera* leaves extracted from the resonance spectra. In this case, leaves at different values of RWC from 1 to 0.75 have been measured. In all cases, the surface density is larger for the PP layer than for the SP layer, which is an expected result due to the fact that the thicknesses of PP and SP layers are similar and the porosity of the SP layer is larger. In addition, the results show that the decrease of the surface density of both layers when the leaves dehydrate is similar, so both follow a similar trend. This suggests that the loss of water in both layers is similar.

Figure 8 shows the surface density of the PP and the SP layers of *V. tinus* leaves extracted from the resonance spectra. In this case, leaves at different values of RWC from 1 to 0.60 have been measured. At full turgor, the surface density is larger for the PP layer than for the SM layer, which is an expected result given the features observed in cross-section images shown in Figure 3A: thickness of both layers is similar, but porosity of the SP layer is much higher. When the leaves loss water, and unlike in *V. vinifera*, the reduction of the surface density is quite different in both layers. Although between RWC 1.0 and 0.95 there are no big differences, for RWC < 0.94, the decrease in the surface density of the PP layer is much more pronounced. Eventually, surface density of both layers becomes similar for RWC < 0.85. CryoSEM images of the cross-section of *V. tinus* at different values of water potential were obtained, with the aim to provide further evidences for the reason for this behavior. Some of these images are shown in Figure 3. At high values of RWC, images show a slimming of the cells of the PP layer while at low RWC values a dramatic contraction of these cells along the direction normal to the leaf plane is observed. This implies a significant PP-cell volume reduction and, hence, a proportional loss of water. On the contrary, loss of cell volume in the SP layer is not so evident. It is clear that there is a reduction of the thickness of the SM layer, but thanks to the larger porosity of this layer, cell flattening is clear, but loss of cell volume (and hence of water) is not so evident as in the PP layer.

## Conclusion

The two-layered model of *L. lucidum, V. tinus* and *V. vinifera* provides a reasonable leaf model approach based on its ability to reproduce all features observed in the ultrasonic leaf resonances and that its consistency with actual leaf structure as revealed by the cryoSEM images. Moreover, comparison of the extracted leaf parameters obtained from this two-layered model and the ultrasonic measurements with data and evidences obtained by alternative methods permit to validate this approach. In particular, overall leaf surface density obtained by the ultrasonic method and from direct measurements agree within the experimental error range. In addition, an indirect verification of the correctness of the approach is provided by the fact that the observed variation of the extracted surface density of the layers of the *L. lucidum* with total leaf thickness are consistent with direct observations of the variation in the thicknesses of each layer.

In *V. vinifera* and *V. tinus*, the technique was used to extract the surface density of each layer for different values of the RWC. These two species present a quite different behavior. While in *V. vinifera* the surface density of both leaf layers decrease is a similar way as RWC decrease, the variation in the surface density of the layers of the *V. tinus* leaves is quite different. While the behavior of the *V. vinifera* leaves is closer to the classical analysis of water relations in leaves where the leaf is considered as a whole, the behavior of *V tinus* leaves shows a clear differential loss of water between the two layers. This unexpected feature is supported by evidences shown in cryoSEM images that show a much larger cell volume loss in the PP than in the SP due to the different cell shape and the presence of a large open porosity in the later.

This differential loss of water in the PP and the SP layers of *V. tinus* leaves pointed out by the ultrasonic technique here presented raise some fundamental questions about how can water potential in different parts of the leaf evolve in a differential way, how equilibrium is reached and what are the mechanisms that make this possible. Loss of water volume in the cells should induce a lower (more negative) osmotic potential (Sanders and Arndt, 2012). The larger volume drop in the cells of PP layer than in the SP layer should, in the absence of any other water potential components, produce a flux of water from the SP layer to the PP layer to recover the equilibrium. It is well documented that plant cells can actively change their osmotic potential by accumulating solutes, in the so-called osmotic adjustment (Boyer et al., 2008; Sanders and Arndt, 2012; Sancho-Knapik et al., 2016). In this situation, a way to compensate for the decrease in water potential in PP due to water loss would be an equivalent increase in solute accumulation (see Hare et al., 1998), but only in SP. While this solute accumulation has been described in photosynthetically active plants in diurnal (Sancho-Knapik et al., 2016) or seasonal cycles (Callister et al., 2008), under the experimental conditions of our measurements it is very unlikely that the equilibrium between PP and SP is restored in this way.

Alternatively, it could be possible that the turgor potential in the PP and SP layers follows a differential evolution so that the equilibrium of water potential between PP and SP is, in this way, kept. In fact, the finding of differences in turgor in different tissues within the same leaf has been described. Fricke (1996) reported such differences between the mesophyll and leaf epidermis as the consequence of “turgor-dependant processes.” Oertli (1986) reported negative turgor pressure in living leaf cells, against previous theoretical assumptions (Tyree, 1976). This fact has been recently revisited (Ding et al., 2014; Yang et al., 2017), and the implications of the ability of living cells to develop negative turgor in the context of water relations of plants under dry conditions has been discussed (Gil-Pelegrín et al., 2017). Assuming differences in the response of cells in SP than in PP, with the development of extra negative potential due to different wall properties in the former may allow reaching equilibrium between both tissues, even with drastic changes in volume (and solute concentration) in PP as compared to SP.

Nevertheless, how the decrease of turgor potential in the cells of the PP layer can be smaller is not clear as the PP-cell volume reduction (and hence the loss of tension in the cell wall) is quite large. These features will be the subject of a future research. In particular, the ultrasonic technique permit to obtain elastic modulus of the PP and SP layers that could provide some insight into the differential evolution of the turgor potential and pressure chamber measurements permit to determine overall leaf water potential.

In summary, this work present a wideband ultrasonic resonant technique for plant leaves that permit to extract the surface density of PP and SP layers of tissue, the technique reveals the capability of the procedure to show the differential growth of thee tissues (*L. lucidum*) and the variation of the mass per area when the leaves loss water (in *V. tinus* and *V. vinifera*). In the particular case of *V. tinus* an unexpected differential evolution of the surface density in PP and SP layers is observed that will be the subject of a future research. This fact reveals the possibilities of the proposed technique that is completely non-destructive and non-invasive and can also be applied *in-vivo*.

## Data Availability Statement

The datasets generated for this study are available on request to the corresponding author.

## Author Contributions

All authors listed have made a substantial, direct and intellectual contribution to the work, and approved it for publication.

## Conflict of Interest

The authors declare that the research was conducted in the absence of any commercial or financial relationships that could be construed as a potential conflict of interest.
